# Oxidative Stress Induced by Reactive Oxygen Species (ROS) and NADPH Oxidase 4 (NOX4) in the Pathogenesis of the Fibrotic Process in Systemic Sclerosis: A Promising Therapeutic Target

**DOI:** 10.3390/jcm10204791

**Published:** 2021-10-19

**Authors:** Sonsoles Piera-Velazquez, Sergio A. Jimenez

**Affiliations:** Jefferson Institute of Molecular Medicine and Scleroderma Center, Thomas Jefferson University, Philadelphia, PA 19107, USA; Sonsoles.piera@jefferson.edu

**Keywords:** fibrosis, systemic sclerosis, oxidative stress, reactive oxygen species, NADPH, NOX4

## Abstract

Numerous clinical and research investigations conducted during the last two decades have implicated excessive oxidative stress caused by high levels of reactive oxygen species (ROS) in the development of the severe and frequently progressive fibrotic process in Systemic Sclerosis (SSc). The role of excessive oxidative stress in SSc pathogenesis has been supported by the demonstration of increased levels of numerous biomarkers, indicative of cellular and molecular oxidative damage in serum, plasma, and other biological fluids from SSc patients, and by the demonstration of elevated production of ROS by various cell types involved in the SSc fibrotic process. However, the precise mechanisms mediating oxidative stress development in SSc and its pathogenetic effects have not been fully elucidated. The participation of the NADPH oxidase NOX4, has been suggested and experimentally supported by the demonstration that SSc dermal fibroblasts display constitutively increased NOX4 expression and that reduction or abrogation of NOX4 effects decreased ROS production and the expression of genes encoding fibrotic proteins. Furthermore, NOX4-stimulated ROS production may be involved in the development of certain endothelial and vascular abnormalities and may even participate in the generation of SSc-specific autoantibodies. Collectively, these observations suggest NOX4 as a novel therapeutic target for SSc.

## 1. Introduction

The most severe clinical and pathologic manifestations of Systemic Sclerosis (SSc) are the result of a fibrotic process characterized by the excessive and often progressive deposition of collagen and other extracellular matrix (ECM) macromolecules in skin and numerous internal organs [1,2,3,4]. Visceral organ fibrosis affecting the lungs and the heart are largely responsible for the high SSc-related mortality [1,2]. However, the precise molecular mechanisms involved in the initiation and progression of the fibrotic process in SSc have not been fully elucidated [5,6]. In 1993, Murrell proposed a novel hypothesis to explain the pathogenesis of tissue fibrosis in various fibrotic disorders, including SSc. The hypothesis suggested that elevated oxygen free radicals caused abnormally increased systemic oxidative stress, which then resulted in cellular and molecular alterations that induced tissue fibrosis [7]. Although reactive oxygen species (ROS) are produced by normal cells and are essential for multiple normal cellular functions, excessive oxidative stress mediated by the uncontrolled production of deleterious ROS has been implicated in the pathogenesis of SSc [7,8,9,10,11,12,13,14,15,16], pulmonary fibrosis [17,18] and several other human fibrotic disorders [19,20].

## 2. Evidence of Oxidative Stress in SSc

There is extensive clinical and experimental evidence demonstrating increased oxidative stress in SSc including elevated serum and plasma levels of malondialdehyde (MDA), asymmetric dimethylarginine (ADMA), 8-isoprostane, and F2 isoprostane; and elevated 8-oxo-7,8-dihydro-2’-deoxyguanosine (8-oxodG), a specific marker of DNA oxidation, in the urine of these patients [21,22,23,24,25,26,27]. It is important to emphasize that the elevated levels of these biomarkers correlate with the extent and severity of cutaneous fibrosis [24,27]. Additionally, elevated levels of 8-isoprostane were described as a biomarker of oxidative stress in SSc-associated and non SSc-related interstitial lung diseases [28,29,30]. The increased level of oxidative stress in patients with SSc-associated Interstitial Lung Disease (SSc-ILD) was also documented by the observation of increased concentrations of 8-epi-PGF2alpha, one of the most abundant isoprostanes present in humans, in bronchoalveolar lavage fluid obtained from patients with SSc fibrosing alveolitis [31].

Extensive experimental evidence supports the role of increased oxidative stress in the development of SSc tissue fibrosis, including the demonstration that fibroblasts isolated from affected SSc skin produce elevated ROS levels compared to normal dermal fibroblasts and that elevated ROS levels induce molecular alterations characteristic of a fibrotic phenotype in these cells in vitro [8,9,10,11,12,13,32,33]. Furthermore, high levels of ROS have been demonstrated in affected and non-affected SSc skin [34]. Similar observations were obtained in a recent study that demonstrated higher levels of intracellular and mitochondrial ROS in SSc dermal fibroblasts and confirmed that the elevated oxidative stress in these cells induced the expression of genes involved in the fibrotic process [35]. This study also showed that SSc skin biopsies displayed evidence of oxidative stress in comparison with normal skin, including the activation of a senescence-like phenotype [35]. Additional experimental evidence implicating excessive oxidative stress and the generation of deleterious ROS in the pathogenesis of SSc was described in a study that demonstrated a pronounced imbalance between the highly elevated oxidative stress levels and the relatively insufficient antioxidant status in SSc [36]. Other evidence of increased oxidative stress in SSc include increased serum levels of N(epsilon)-(hexanoyl)lysine [37], elevated serum levels of heat shock protein 70 [38], abnormalities of erythrocyte membrane fluidity and lipid oxidation status [39], and increased serum pentraxin 3 levels [40]. The most common oxidative stress biomarkers that are elevated in various biological fluids from SSc patients are shown in Table 1.

Although there are numerous cellular sources of ROS production, several cell types intimately involved in SSc pathogenesis besides fibroblasts and endothelial cells have been shown to contain elevated ROS levels. For example, it has been described that monocytes isolated from SSc patients spontaneously release increased amounts of certain ROS [41], and that SSc neutrophils and T-lymphocytes are also capable of ROS production [42,43].

## 3. Role of ROS in SSc Pathogenesis

Under physiological conditions, ROS participate in multiple cellular functions and signaling pathways and exert these effects by regulating the expression of numerous genes [44,45,46,47,48]. However, in pathological states, elevated ROS levels can induce oxidative stress damage to proteins, lipids, and DNA, as well as activate various deleterious redox-sensitive pathological cell signaling pathways and, therefore, they have been shown to participate in the development or progression of a wide spectrum of human diseases [49,50].

Following Murrell’s provocative hypothesis [7], numerous studies examined the role of ROS and free radical-induced oxidative stress in the initiation and progression of exaggerated fibrogenesis in SSc and initiated the exploration of the potential mechanisms involved [8,9,10,11,12,13,14,15,16]. Regarding the fibrotic process, the functional relevance of the elevated oxidative stress components in the circulation of SSc patients was evidenced in one study showing that sera from patients with SSc pulmonary hypertension caused oxidative stress-induced stimulation of collagen synthesis in human pulmonary smooth muscle cells in vitro [51], and the demonstration by Sambo et al. [8], in which the increased levels of ROS in SSc dermal fibroblasts resulted in the establishment of a pro-fibrotic phenotype in these cells. More recently, extensive investigations have assessed cutaneous oxidative stress in SSc, employing novel quantitative methods including fluorescence spectroscopy and tissue oxygenation spectrophotometry of affected SSc skin [52,53]. These studies suggested that the structural and functional microvascular abnormalities characteristic of the SSc vasculopathy cause tissue hypoxia and that as a result, there is increased oxidative stress in the affected tissues [52,53]. Furthermore, several studies have indicated that the increased oxidative stress induced by ROS alterations is reflected in the extent and severity of cutaneous fibrotic and skin thickening in these patients [24,27,54,55].

## 4. Molecular Mechanisms of ROS-Induced Fibrosis in SSc

**Figure 1:**
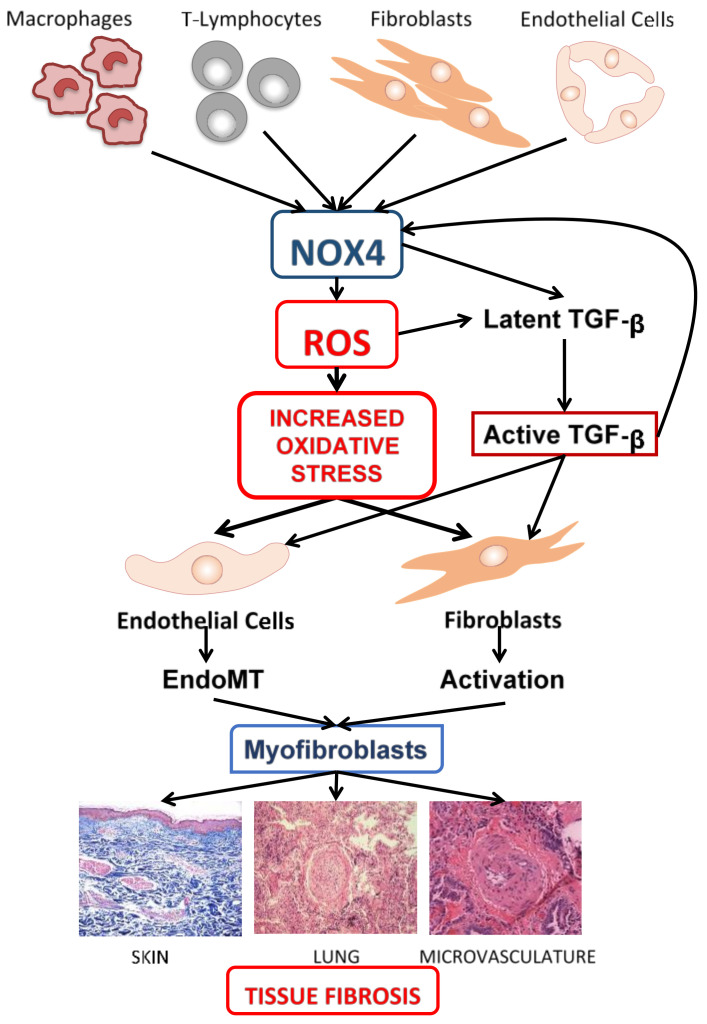
Schematic diagram depicting the mechanisms involved in the generation of increased oxidative stress and in the induction of SSc-associated tissue fibrosis. Numerous cell types relevant to SSc pathogenesis produce ROS and express NOX4. Stimulation of transcription of the NOX4 gene leads to increased NOX4 and elevated ROS production. These cells produce elevated ROS levels that are capable of inducing oxidative stress in various target cells as well as phenotypic changes in fibroblasts and endothelial cells, causing their conversion into activated myofibroblasts. Additionally, increased ROS production leads to release of TGF-β from the Latent TGF-β protein complex in the extracellular matrix, followed by its activation and activated TGF-β can then establish a paracrine pathway by causing further stimulation of NOX4 production. Activated TGF-β also directly stimulates the fibrotic process by inducing myofibroblast expansion through EndoMT and fibroblast activation. The activation of the ROS/NOX4 pathway leads to tissue fibrosis as illustrated in histopathological images of SSc skin, lung, and microvasculature.

The molecular mechanisms of ROS stimulation of the fibrotic process are highly complex and involve multiple molecular pathways, including the ROS-mediated activation of latent TGF-β1 and the phenotypic conversion of quiescent fibroblasts and endothelial cells into profibrotic myofibroblasts. The newly generated myofibroblasts are highly active mesenchymal cells capable of producing large amounts of interstitial fibrillar collagens and other fibrotic proteins. The elevated production of these proteins by the activated myofibroblasts and their exaggerated accumulation in the interstitial space of the affected organs results in severe and often progressive fibrotic alterations characteristic of SSc, as illustrated in Figure 1.

Owing to the potential therapeutic relevance of ROS-mediated pro-fibrotic effects there have been numerous studies focused on the elucidation of the molecular pathways involved. Among these, a recent study showed that ROS induced a potent transcriptional inhibition of the anti-Wnt protein, Wnt inhibitory factor 1 (WIF-1), leading to activation of Wnt pathway-induced tissue fibrosis [56]. Other investigations examining the ROS-mediated profibrotic pathways included a study that demonstrated a dose dependent abrogation of the increased production and secretion of type I collagen and fibronectin characteristic of SSc fibroblasts following their in vitro exposure to the potent antioxidant, epigallocatechin-3-gallate (EGCG) [57]. EGCG also caused a marked inhibition of intracellular ROS and reduction in the expression of genes encoding fibrosis-associated proteins and abrogated several molecular pathways involved in the fibrotic process, including the signalling mediated by intracellular ERK1/2 kinase [57]. In similar investigations Tsou et al. described a novel mechanism by which ROS may promote the establishment of a fibrotic phenotype in SSc fibroblasts. This pathway involves the oxidative inactivation of protein tyrosine phosphatase 1B (PTP1B), mediated by higher levels of ROS [58]. This study also showed that ROS production was significantly higher in SSc fibroblasts, whereas PTP1B activity was significantly reduced in these cells. The role of PTP1B on the regulation of the fibrotic process was confirmed by the demonstration that reduction of PTP1B expression in normal dermal fibroblasts caused increased type I collagen production by these cells. Furthermore, restoration of low PTP1B activity by scavenging ROS with the potent thiol antioxidant N-acetyl cysteine (NAC) caused a marked reduction in type I collagen production by SSc dermal fibroblasts [58]. Similar results were obtained by Mancini et al. [35] who demonstrated that treatment of cultured SSc fibroblasts with NAC reduced the expression of numerous genes encoding tissue fibrosis-associated molecules, including COL1A1 and COL1A2, as well as other profibrotic and inflammatory molecules such as IL6, ICAM1 and TGFβ3 [35].

## 5. Possible Role of ROS in the Induction of SSc Autoantibodies

Besides ROS stimulation of the fibrotic process in SSc, there are some novel studies supporting the possibility that elevated ROS may also play a role in the development of the autoimmune component in SSc. In one of these studies, it has been shown that ROS may induce abnormal oxidation of DNA-topoisomerase-1 and that this molecular/structural modification may result in an increase in its antigenicity and, therefore, may be responsible for the production of anti-DNA-topoisomerase (Scl-70) autoantibodies, the prototypic autoantibody marker in diffuse SSc [59]. The potential role of ROS in the generation of various autoantibodies has been described in other autoimmune diseases including Rheumatoid Arthritis and Systemic Lupus [60]. These studies collectively provide support for the participation of ROS in the generation of disease-specific autoantibodies and suggest that elevated ROS may also be involved in some of the immunologic alterations, characteristic of SSc.

## 6. Molecular Mechanisms of ROS Generation: NADPH Oxidases (NOX)

There are multiple biochemical pathways and enzymatic sources that are capable of intracellular ROS production. The most important include ROS production inside mitochondria, and ROS generation as a result of the activity of numerous intracellular enzymes, including lipoxygenases, cyclooxygenases, and xanthine oxidases [44,45,46,47,48]. However, extensive studies have shown that the principal intracellular source of ROS production is the activation of nicotinamide adenine dinucleotide phosphate (NADPH) oxidase pathways or NOX enzymes [61,62,63,64,65]. The crucial role of NOX in normal cellular physiology is evidenced by the remarkable increase in the number of NOX isoforms during eukaryotic evolution and their striking conservation through multiple species [66]. Currently, seven distinct NOX isoforms have been identified in humans and there are substantial differences in their tissue distribution [67]. However, extensive studies have shown that several of the NOX enzymes display prominent expression in the vasculature and in other tissues and cells of substantial relevance to the pathophysiology of fibrotic disorders including SSc and pulmonary fibrosis [68,69,70,71,72,73,74]. The three most important NOX isoforms related to SSc are NOX1, NOX2, and NOX4. NOX1 is abundantly expressed in endothelial cells and vascular smooth muscle cells. NOX2 is the classic inflammatory isoform found in neutrophils and macrophages but is also expressed in B-lymphocytes and in endothelial cells. NOX4 was first discovered and is very highly expressed in the kidneys [75], however, it displays very high expression in fibroblasts, smooth muscle cells, and microvascular endothelial cells, as well as in lung epithelial cells [68,69,70,71,72,73,74,76].

## 7. Role of NOX4 in Tissue Fibrosis

Numerous recent studies have indicated that NOX4 may play a crucial role in the initiation, establishment and progression of tissue fibrosis [32,77,78,79], and that these effects are mediated by various growth factors and several other growth factor-related molecules. Indeed, multiple growth factors which participate in the development of pathological fibrotic processes, most prominently, TGF-β [80,81], have been shown to modulate the expression of NOX, and in particular, that of NOX4. Stimulation of NOX4 expression by TGF-β has been demonstrated in various cells and different tissues including dermal, pulmonary, and cardiac fibroblasts, pulmonary artery smooth muscle cells, and hepatocytes [11,13,32,82,83,84,85,86]. It has also been shown that these effects are mediated by a far-upstream AP-1/Smad regulatory element in the NOX4 gene promoter [87]. PDGF, angiotensin II, endothelin-1, and insulin-like growth factor have also been shown to induce increased NOX4 expression [88,89,90,91]. On the other hand, NOX4 has been identified to be involved in TGF-β-induced generation of dermal, pulmonary, cardiac, liver, and renal myofibroblasts in vitro [11,13,32,82,83,84,85,86]. Of relevance to the role of NOX4 in the fibrotic process of SSc are recent studies demonstrating that ROS were capable of inducing the phenotypic conversion of quiescent fibroblasts and endothelial cells into activated myofibroblasts through fibroblast-myofibroblast and endothelial to mesenchymal transition (EndoMT) phenotypic changes, respectively [92,93,94,95,96]. A related investigation demonstrated that these phenotypic change modulatory events were likely mediated by TGF-β1 in endothelial cells [95].

## 8. Regulation of NOX4 Activity

The mechanisms involved in the regulation of NOX activity are quite complex and vary depending on the specific cellular and functional context. However, in contrast with other NOX enzymes, NOX4 does not require other protein subunits for its activity and, therefore, the extent and intensity of its effects are entirely dependent on the levels of expression of its corresponding gene [97]. Given the important functions of NOX4 in a variety of physiological processes and in the pathogenesis of numerous diseases, the intimate mechanisms involved in its regulation have been the focus of intense investigation, and particular interest has been placed on the regulation of NOX4 expression by TGF-β and other growth factors [82,83,84,85,95]. Although the exact mechanisms involved in the regulation of NOX4 levels in normal cells are becoming unveiled, the possible alterations responsible for the constitutive elevation in NOX4 levels and activity in SSc cells have not been fully revealed. An extensive study examined NOX4 expression in normal dermal fibroblasts and showed that TGF-β1 induced a greater than three-fold stimulation of NOX4 transcript levels in normal dermal fibroblasts and that the stimulation of NOX4 gene expression induced by TGF-β was almost completely abrogated by siRNA inhibition of PKC-δ. Furthermore, Smad2/3 were also shown to be involved in this regulation since the specific Smad 2/3 inhibitor SB431542 caused essentially complete abrogation of TGF-β1 stimulation of NOX4 expression. These results were confirmed at the protein level assessed by Western blots for NOX4 [32].

## 9. NOX4 Expression in Normal and SSc Dermal Fibroblasts and NOX4 Effects on Production of Fibrotic Molecules

Numerous studies have examined NOX4 expression levels in cultured normal and SSc human dermal fibroblasts and characterized its effects on molecular pathways involved in the exaggerated production and accumulation of fibrotic extracellular matrix (ECM) proteins in affected SSc tissues [8,11,32,58,77,98]. One extensive study showed that NOX4 was constitutively expressed by cultured normal human dermal fibroblasts and that its expression was significantly higher in cultured fibroblasts isolated from affected SSc skin. The effects of NOX4 on ROS production by dermal fibroblasts were validated employing an in vitro analysis of cellular fluorescence using carboxy-H_2_DCFDA, a compound that permeates live cells and that in the presence of ROS becomes oxidized and emits a bright fluorescence that can be quantitatively assessed. In order to analyze NOX4-specific effects, normal dermal fibroblasts were treated with TGF-β1 to stimulate NOX4 expression, followed by incubation with GKT137831, a highly specific dual inhibitor of NOX4/NOX1. The results confirmed the expected increase in ROS production induced by TGF-β1 and demonstrated a marked reduction in TGF-β-stimulated ROS production by the specific NOX4/NOX1 inhibitor. To directly examine the role of NOX4 on the expression of genes encoding ECM molecules and on the levels of production of type I collagen in SSc, cultured SSc dermal fibroblasts were transfected with specific siRNA against NOX4 resulting in a 60% reduction of NOX4 mRNA. The NOX4 RNA decrease resulted in a significant reduction in ROS production and, more importantly, caused a 20%–30% decrease in type I collagen protein levels secreted into the culture media of the SSc dermal fibroblasts. The results of this extensive study confirmed increased NOX4 expression and ROS production by SSc dermal fibroblasts and showed that stimulation of ROS production by NOX4 mediated potent pro-fibrotic effects, since a significant reduction of type I collagen production was observed as a result of NOX4 siRNA inhibition in these cells [32].

## 10. NOX4 and Oxidative Stress Induction of EndoMT

Numerous studies have demonstrated that endothelial cells are capable of a remarkable change in their specific cellular phenotype and that under certain conditions they undergo a conversion into myofibroblastic mesenchymal cells through the complex process of EndoMT. During EndoMT, endothelial cells adopt a myofibroblast cell morphology and initiate the expression and production of mesenchymal cell-specific proteins, including α-smooth muscle actin, extra domain A fibronectin, and fibrillar type I and type III collagens [99,100,101]. Based on these studies, it has recently been suggested that EndoMT may play a crucial role in the pathogenesis of fibrotic disorders, including SSc [102,103,104]. Oxidative stress and ROS derived from the activation of NOX pathways have been recently shown to be important inducers of EndoMT and it has been suggested that this mechanism may play a role in the profibrotic alterations mediated by ROS [95,96]. The most relevant NOX species regarding endothelial cell functions and pathology are NOX2 and NOX4 isoforms that are present at high levels in the vascular endothelial compartment. Although the role of NOX4 in EndoMT has not been directly examined, given that NOX4 is an important downstream mediator of TGF-β-induced effects, it has been recently suggested that it may also induce EndoMT. Furthermore, in relation to ROS-mediated induction of EndoMT, it was demonstrated that NAC abrogated endotoxin-induced EndoMT in human umbilical vein endothelial cells [105]. Thus, there is strong experimental evidence to support the crucial role of ROS and NOX in the initiation and/or progression of EndoMT and therefore provide an additional and highly relevant mechanism supporting the antifibrotic effects of NOX4 inhibition in SSc.

## 11. Potential Therapeutic Implications of ROS/NOX4 Inhibition for SSc Tissue Fibrosis

As discussed extensively in previous sections, increased oxidative stress caused by excessive and unbalanced generation of ROS in affected tissues contributes to the development and progression of the fibrotic process in SSc. Furthermore, the elevated ROS levels in SSc are mediated by high levels of NOX4 activity. Based on this concept, it has been postulated that assessment of ROS production and of the main enzymatic pathways involved in ROS generation and production may allow to predict the clinical evolution of SSc and may also indicate a favorable therapeutic response of the disease process [105]. Accordingly, it has been suggested that the administration of antioxidants and NOX4 inhibitors may be considered effective disease-modifying therapeutic interventions for SSc and other fibrotic disorders, including pulmonary and liver fibrosis [106,107,108,109,110,111,112,113,114,115,116,117,118,119].

Regarding SSc, there are few clinical trials that have actually tested the therapeutic effectiveness of antioxidant therapies for the disease [120,121,122,123,124]. Among these, a study assessed the effects of alpha-tocopherol combined with ascorbic acid on skin thickening and lung function in patients with early diffuse SSc with positive anti-topoisomerase-I antibody and decreased DLCO (75% of predicted or lower) treated with monthly intravenous cyclophosphamide (500 mg/m^2^ of body surface). One group of six patients received cyclophosphamide plus the antioxidants, and the comparison group of seven patients received only cyclophosphamide. After six months of therapy, patients treated with cyclophosphamide plus antioxidants had a significantly lower rate of progression of skin involvement and their DLCO improved slightly, although the improvement was not statistically significant compared to patients treated with cyclophosphamide without antioxidants, in whom the pulmonary function studies showed a small deterioration [120]. Another report described a double-blind placebo-controlled trial of a combination of antioxidant micronutrients plus allopurinol in 33 patients with limited cutaneous SSc. The results failed to show any evidence of decreased oxidative stress as the levels of various oxidative damage biomarkers were similar after 10 weeks of therapy compared with the results in samples from the placebo group [121].

Several studies examined the effects of NAC on cutaneous and pulmonary fibrotic involvement in SSc [122,123,124]. Furst et al. [122], performed a parallel, double-blind, placebo-controlled study in 22 patients with progressive SSc. The treatment group received NAC administered orally for 1 year and its effects were assessed at 6 and 12 months. An extensive analysis and testing performed in this study failed to show any significant antifibrotic effects of NAC [122]. Another study described a retrospective analysis in patients who had received high dose NAC intravenously for treatment of Raynaud’s Phenomenon given for 5 h every 14 days for 24 months. In this study, there was a modest, although significant improvement in pulmonary function tests (FVC and DLCO) and also stabilization of pulmonary fibrosis as assessed by semiquantitative high resolution chest CAT score [123]. A more recent study examined the effects of oral NAC on pulmonary function tests in 25 patients with diffuse SSc without pulmonary involvement. The study was a randomized placebo controlled double-blind trail. Twenty-five patients were studied (13 on placebo and 12 on 1200 mg daily oral NAC) for 24 weeks. The results showed that NAC had no effect on DLCO and several clinical parameters compared with placebo [124].

The few studies that examined oxidative stress inhibitors in SSc patients are summarized in Table 2 [120,121,122,123,124]. It should be emphasized, however, that there are no clinical studies that examined directly the effects of NOX4 inhibition despite the extensive evidence that NOX4 plays a most important role in the generation of oxidative stress. In this regard and of potential therapeutic relevance for SSc patients are studies that described the synthesis of novel and highly selective NOX4 inhibitors displaying good oral absorption [125]. Although these compounds have been shown to be potent inhibitors of NOX4 in vitro and may also effectively reduce oxidative stress in vivo, to date, they have not been tested clinically.

Overall, the results of the clinical studies discussed above regarding the therapeutic effects of antioxidants for SSc have been negative or non-conclusive. Therefore, it will be necessary to perform further studies including well controlled placebo matched clinical trials to document any beneficial effects of antioxidant/NOX4 inhibitory therapeutic interventions on the development or progression of the fibrotic process in SSc. Furthermore, the participation of ROS/NOX occurs most likely during the earliest stages of development of the fibrotic process in SSc, therefore, antioxidant drugs should be administrated very early in the development of tissue fibrosis to increase their possible beneficial therapeutic effects. In addition, it should be emphasized that the combined use of antioxidant drugs that exert their effects through different molecular mechanisms may further result in improved disease-modifying effects for this disabling and frequently fatal disease.

## 12. Conclusions

This review examines the abundant scientific literature and numerous recent experimental studies that provide strong evidence to indicate that exaggerated oxidative stress caused by increased levels of ROS is involved in the development of the SSc-associated fibrotic process and that NOX4 is the most important source of increased ROS in SSc [32,33,34]. Furthermore, we discuss evidence demonstrating that NOX4 expression is stimulated by TGF-β, the most potent profibrotic growth factor in SSc [80,81]. Therefore, it is likely that NOX4 plays an important role in the pathogenesis of SSc fibrosis. Based on the available evidence discussed in this Review, it is suggested that the successful inhibition of NOX4 as one of the principal sources of oxidative stress generation should be further studied as a highly relevant therapeutic target for the disease. However, despite a solid scientific rationale for the role of oxidative stress in SSc, the administration of antioxidant therapeutic interventions to SSc patients has been quite disappointing and has failed to consistently result in beneficial effects [10,11,12,13,14,15,16,121,122,123,124]. Amongst the possible explanations for this discrepancy may be that the disease was at an advanced stage at initiation of the antioxidant treatment, as well as the less-than-optimal duration of the therapeutic intervention. Therefore, it is suggested that in order to show detectable and reproducible beneficial effects on the clinical manifestations of the fibrotic process in SSc, the therapy aimed at the reduction of the excessive oxidative stress must be initiated early in the course of the disease process, prior to the establishment of irreversible tissue fibrotic alterations, and should be administered at a high dosage and maintained for a prolonged duration. Thus, although there is strong experimental evidence supporting the role of excessive oxidative stress in the development of the SSc-associated fibrotic process, further and well controlled clinical studies will be necessary to confirm the therapeutic beneficial effects of interventions capable of reducing the deleterious effects of elevated oxidative stress in SSc.

## Figures and Tables

**Table 1:** Biomarkers of Oxidative Stress in Systemic Sclerosis.

Biomarker	Origin	Alteration in SSc	Ref.
Malondialdehyde (MDA)	Specific result of fatty acids peroxidation	High plasma and serum levels	[21,27]
8-oxo-dihydro-deoxyguanosine	Product of endogenous DNA oxidative damage	High levels in urine	[26]
F2-isoprostane	Product of non-cyclooxygenase free radical-mediated arachidonic acid peroxidation	Associated with a fibrotic phenotype	[22,23,24,25,27]
8epi-PGF2 α isoprostane	ROS oxidation of tissue phospholipids	Elevated in BAL fluid in SSc-associated ILD, correlates with ILD severity	[24,31]
N (epsilon)-(hexanoyl) lysine	Protein oxidation in numerous cell types	Increased in Serum	[37]
HSP 70	Protein present in numerous cell types	Elevated serum levels associated with tissue fibrosis and vasculopathy	[38]
Abnormal RBC membrane lipid oxidation	RBC membrane lipids	Abnormal RBC membrane fluidity	[39]
Pentraxin 3	Released from Inflammatory cells	Increased	[40]
Advanced Oxidation Protein Products	ROS-induced cellular senescence and oxidation of proteins	Indicate oxidative protein damage	[35,36]

BAL: Broncho-alveolar lavage; HSP: Heat Shock Protein; ILD: Interstitial lung disease; RBC: Red blood cell.

**Table 2:** Clinical Studies of Oxidative Stress Inhibitors in SSc Patients.

Inhibitor	SSc Patient Population	Clinical Trial	Clinical Effects	Ref.
α-tocopherol and ascorbic acid	Early Diffuse SSc patients with reduced DLCO and +Scl-70	6 month treatment +/− cyclophosphamide	Reduced skin thickening progression rate and improved DLCO (not significantly)	[120]
Combination of antioxidant micronutrients +Allopurinol	Limited Cutaneous SSc patients	10 weeks treatment	NO beneficial effects	[121]
N-acetyl cysteine (NAC)	Progressive SSc patients	1 year treatment +/− oral NAC	No change in skin and lung fibrotic measures	[122]
	Diffuse and Limited SSc	Retrospective 4 months study of IV NAC every 4 days	Improvement in lung function studies	[123]
	Diffuse SSc	24 wk double-blind randomized placebo study	No effect on DLCO	[124]

## Data Availability

Not applicable.
